# Applications and analysis of hydrolysates in animal cell culture

**DOI:** 10.1186/s40643-021-00443-w

**Published:** 2021-09-28

**Authors:** Yin Ying Ho, Hao Kim Lu, Zhi Feng Sherman Lim, Hao Wei Lim, Ying Swan Ho, Say Kong Ng

**Affiliations:** Bioprocessing Technology Institute, Agency for Science, Technology, and Research (A*STAR), 20 Biopolis Way, #06-01 Centros, Singapore, 138668 Singapore

**Keywords:** Hydrolysates, Animal cells, Cell culture

## Abstract

Animal cells are used in the manufacturing of complex biotherapeutic products since the 1980s. From its initial uses in biological research to its current importance in the biopharmaceutical industry, many types of culture media were developed: from serum-based media to serum-free to protein-free chemically defined media. The cultivation of animal cells economically has become the ultimate goal in the field of biomanufacturing. Serum serves as a source of amino acids, lipids, proteins and most importantly growth factors and hormones, which are essential for many cell types. However, the use of serum is unfavorable due to its high price tag, increased lot-to-lot variations and potential risk of microbial contamination. Efforts are progressively being made to replace serum with recombinant proteins such as growth factors, cytokines and hormones, as well as supplementation with lipids, vitamins, trace elements and hydrolysates. While hydrolysates are more complex, they provide a diverse source of nutrients to animal cells, with potential beneficial effects beyond the nutritional value. In this review, we discuss the use of hydrolysates in animal cell culture and briefly cover the composition of hydrolysates, mode of action and potential contaminants with some perspectives on its potential role in animal cell culture media formulations in the future.

## Introduction

Biopharmaceutical products are playing an increasingly important role in the management and treatment of diseases ranging from infections to enzymatic deficiencies and cancers. According to a recent market report published by Mordor Intelligence, the biopharmaceutical market has reached approximately $325.17 billion in 2020 with a compound annual growth rate (CAGR) of 7.32% from 2021 to 2026, that is a revenue of $496.71 billion in 2026 (Mordor Intelligence 2021). A prominent example of a biopharmaceutical product is monoclonal antibodies (mAbs), which are becoming the standard of care for indications in oncology and inflammation. Despite the Covid-19 pandemic, mAb therapeutic sales are expected to grow to $114.43 billion in 2021 and reach $179.56 billion in 2025, a CAGR of 11.9% (The Business Research Company 2021). Currently, the majority of the biopharmaceutical products are made using animal cell systems because of their ability to produce correctly folded and fully glycosylated proteins that other simpler organisms cannot replicate (Li et al. 2010). The ultimate goal of process development in animal cell culture is to increase product quality and yield while reducing cost. However, despite constant bioprocessing improvement in both upstream and downstream operations, high production cost remains the bottleneck that limits demand (Xu et al. 2020). The cell culture medium is an important component of raw materials that contributes significantly to the cost of production. Based on the type of supplements added, animal cell culture media can be broadly described as chemically defined medium, protein-free medium, animal component-free medium and serum-containing medium (Yao and Asayama 2017). Almost all cell lines were originally developed in media that contain animal serum and later adapted to serum-free media. Serum-supplemented medium is effective for a variety of cell types as it contains an abundance of known and unknown factors such as growth factors, hormones and lipid components that are necessary for the survival and proliferation of cells (O’Flaherty and Bergin 2020). However, the use of animal serum in media not only increases the risk of contamination with infectious agents, but also contributes significantly to the cost of production (O’Flaherty and Bergin 2020). Researchers are increasingly moving away from the use of animal serum and into animal-component-free media. The removal of serum not only improves production consistency, resulting in less batch-to-batch variability, but also reduces downstream processing time and cost. Many scientists have embarked on the journey in exploring alternatives to serum, to discover simple, low-cost and highly reproducible media for culturing animal cells. Current alternatives to serum include the use of a combination of recombinant proteins (e.g., insulin and other growth factors), hormones (e.g., hydrocortisone), lipids, and hydrolysates.

Hydrolysates are the products of plant (soy, pea, rice, rapeseed, etc.) or animal (chicken, pork, fish, etc.) proteins after hydrolysis by acid, alkali, enzymes and fermentation processes. When a predominantly protein starting material is used to produce the hydrolysate, the product may be described as a protein hydrolysate, for example, rice protein hydrolysate and rapeseed protein hydrolysate. Hydrolysates often contain a mixture of peptides, amino acids, minerals, carbohydrates, lipids and proteins that are similar to the raw input material. Since the late 1970s, chicken and fish-derived hydrolysates, have been used as serum replacements for animal cell culture (Mizrahi 1977). However, as animal-derived hydrolysate would encounter the same issues as using animal serum, the use of plant-based hydrolysates such as those derived from soy, rice and cottonseed proteins have gained popularity. The relatively low cost of plant-based hydrolysates makes them attractive as serum replacement components for large-scale protein production. However, given hydrolysate products are not fully characterized, further understanding of their components and how these can influence cell growth and maintenance is key to their success as potential serum replacement components. In this review, we will describe the history of animal cell culture, including examples of animal cell cultures used for the production of biotherapeutics in the presence or absence of serum, the history of the use of hydrolysates in animal cell cultures for biotherapeutics and new modalities such as cultured meat production. The review will also describe the compositions of hydrolysate products, their modes of action and potential contaminants with some views on their future use in animal cell cultures.

## The advent of animal cell culture and its use in biotherapeutics production

The ability to culture and study cells outside of the animal/host’s body in the last 150 years has led to the development of modern science and medicine that we know today. The first attempt of cell culture dates back to the late nineteenth century when Wilhelm Roux demonstrated that it is possible to maintain living cells of the neural plate of the chick embryo in saline for a few days (Rodríguez-Hernandez et al. 2014). More successful attempts at tissue culture were achieved when a small amount of serum or lymph was added to the culture. In 1907, Ross Granville Harrison developed a reproducible technique of tissue culture by placing the neural tube frog embryo into a drop of fresh lymph and inverted the coverslip, generating the first hanging drop culture technique (Harrison et al. 1907). The first tissue culture of animal cells was described a few years later by Montrose Burrows and Alexis Carrel using culture media that contains animal plasma (Carrel and Burrows 1911a, b). They also demonstrated that these cell lines can be maintained in culture for several months and that the cells could grow extensively in media containing plasma of other mammals including rabbits, dogs, or humans (Carrel and Burrows 1911a, b). However, it was not until 1948 that the first cell line, L929, a mouse connective tissue fibroblast was established (Earle et al. 1943). In 1951, an aggressive adenocarcinoma of the cervix gave rise to HeLa cell (Gey 1952), one of the most commonly used human cell lines for medical research. The establishment of cell lines has not only contributed to our understanding of many diseases, but it has also become an invaluable tool for the production of biotherapeutics, ranging from recombinant protein biologics to virus production for gene therapy (Table 1).

**Table 1:** Establishment of important cell lines for research and biopharmaceutical manufacturing in order of year of origin

Name	Species and tissue	Morphology	Culture in serum-free media	Year of origin
L929	Mouse connective tissue	Fibroblast	No	1948; Earle et al. (1943)
HeLa	Human cervix	Epithelial	Yes	1951; Gey (1952)
CHO	Chinese hamster ovary	Epithelial-like	Yes	1957; Tjio and Puck (1958)
Baby hamster kidney (BHK) cells	Hamster kidney cells	Fibroblast	Yes	1961; Hernandez and Brown (2010)
Vero	African green monkey kidney	Fibroblast	Yes	1962; Yasumura and Kawakita (1963)
3T3	Mouse embryo	Fibroblast	No	1962; Todaro and Green (1963)
U-2 OS	Human osteosarcoma	Epithelial	No	1964; Ponten and Saksela (1967)
MCF 7	Human adenocarcinoma	Epithelial	No	1970; Soule et al. (1973)
HT1080	Human fibrosarcoma	Epithelial	No	1974; Rasheed et al. (1974)
Sp2/0	Fused BALB/c mouse spleen cell and mouse myeloma P3X63Ag8	Lymphoblast	Yes	1975; KÖHler and Milstein (1975)
Caco-2	Human colorectal adenocarcinoma	Epithelial	No	1977; Fogh et al. (1977)
Jurkat	Human T cell leukemia	Lymphoblast	No	1977; Schneider, et al. (1977)
HEK293	Human embryonic kidney	Epithelial	Yes	1977; Graham et al. (1977)
THP-1	Human monocytic leukemia	Monocytic	Yes	1980; Tsuchiya et al. (1980)
NS0	Murine myeloma	Lymphoblast	Yes	1981; Galfrè and Milstein (1981)
PER.C6	Human embryonic retinal cells	Epithelial	Yes	1999; Havenga et al. (2008)
HKB-11	human hybrid kidney/B cell	Epithelial	Yes	2001; Cho et al. (2003)
AGE1.CR	Duck embryo	Fibroblast	Yes	2007; Jordan et al. (2009)
CEVEC’s amniocyte production (CAP)	Primary human amniocytes	Epithelial	Yes	2011; Wölfel et al. (2011)

In 1975, the first large-scale production of antibodies was described by Georges Köhler and Cesar Milstein (1975). They combined an antibody-producing B cell with a myeloma cell line; the resulting hybridoma acquired the ability to divide rapidly and at the same time produce the antibody. Since then, antibodies and other recombinant proteins have become the predominant products in the biopharmaceutical industry. Animal cells are the preferred choice for biotherapeutic manufacturing as these cell lines are capable of producing complex proteins with post-translational modifications similar to those produced in humans (Durocher and Butler 2009; Ghaderi et al. 2012; Sha et al. 2016). In addition, animal cells can secrete proteins efficiently and at high titers, facilitating protein production and purification (Dumont et al. 2016).

In 1986, the first therapeutic protein, the human plasminogen activator, produced in the Chinese Hamster Ovary (CHO) cell line was approved by the US Food and Drug Administration (FDA) (Wurm 2004). Since then, the CHO cell system has continued to be the leading cell line of choice for human therapeutic production, accounting for ~ 70% of recombinant protein production in the 2000s (Jayapal et al. 2007). The popularity of CHO cells and CHO variants can be attributed to the following reasons. Firstly, CHO cells can grow in serum-free chemically defined media in suspension culture. The removal of serum reduces lot-to-lot variability and produces an improved safety profile for the therapeutic protein, in comparison to therapeutic protein produced in media containing animal-derived proteins. Secondly, since the first approved therapeutic protein was produced in CHO cells and with more than three decades of safety data, it may be easier to obtain regulatory and consumer acceptance. Thirdly, being a hamster-derived cell line, CHO cells are less susceptible to certain human viral infections, likely because some of the human viral entry genes are not expressed in these cells (Xu et al. 2011). Finally, a powerful system of gene amplification, such as dihydrofolate reductase (DHFR) or glutamine synthetase (GS) has previously been described in CHO cells. These gene amplification systems can improve recombinant protein yield and specific productivity, which was previously an issue in other animal cell lines (Kim et al. 2012; Dumont et al. 2016).

Despite the popularity of the CHO cell line in protein therapeutic production, it is unable to produce the full range of glycosylation found in humans (Patnaik and Stanley 2006), while it also produces rodent-specific glycan structures that may lead to increased immunogenicity in humans (Bosques et al. 2010; Ghaderi et al. 2012). This has led to the use of human-derived cell lines such as HEK293 (Human Embryonic Kidney cells) for therapeutic protein production. HEK293 can be easily grown in suspension serum-free culture; the cells also grow rapidly, possess superior transfection efficiency and express high levels of protein (Vink et al. 2014). Other variants of this cell line, such as HEK293-T, express an allele of the simian virus 40 large T antigen and are capable of expressing high titers of viral gene vectors (Yamaguchi et al. 2003). HEK293-T cells are often used for the production of retroviral vectors for cell and gene therapy (Ferreira et al. 2019).

While both CHO and HEK293 are capable of growing in serum-free media, we noted that both cell lines are immortalized and may have accumulated undefined mutations to survive without serum. Although HEK293 became an immortalized cell line after being transformed with sheared adenovirus-5 DNA (Kovesdi and Hedley 2010), CHO cells were immortalized spontaneously (Wurm and Wurm 2017). Some other cell lines have not been reported to be capable of serum-free culture; for example, the commonly used human Colorectal Adenocarcinoma cell line, Caco-2, requires the use of serum for survival and maintenance (Table 1). Yao and Asayama (2017) have published a detailed review on the history, characteristics and current issues the community is facing over animal cell culture media . The development of serum-free media has many considerations such as defining the composition and concentration of various components in the media; it has been a subject of research for many decades with limited success.

## The use of hydrolysate in animal cell culture

Although animal serum is capable of supporting the growth of almost all cell lines (Zheng et al. 2006), the risks associated with its use outweigh the benefits. Furthermore, the US FDA and European Medicine Agency have tightened their rules on the use of animal-derived components in commercial protein production out of concerns for patient safety (Merten 2002; Siemensma et al. 2010). It is therefore important to investigate the essential components contain in serum that supports the growth of animal cells. Despite the availability of some animal-component-free media for selected cell lines, such media is not available for all cells, and their development often requires an enormous amount of time and money (Ballez et al. 2004). Protein digests from different types of raw materials, termed hydrolysates, have been proposed as alternatives to components in serum.

**Fig. 1:**
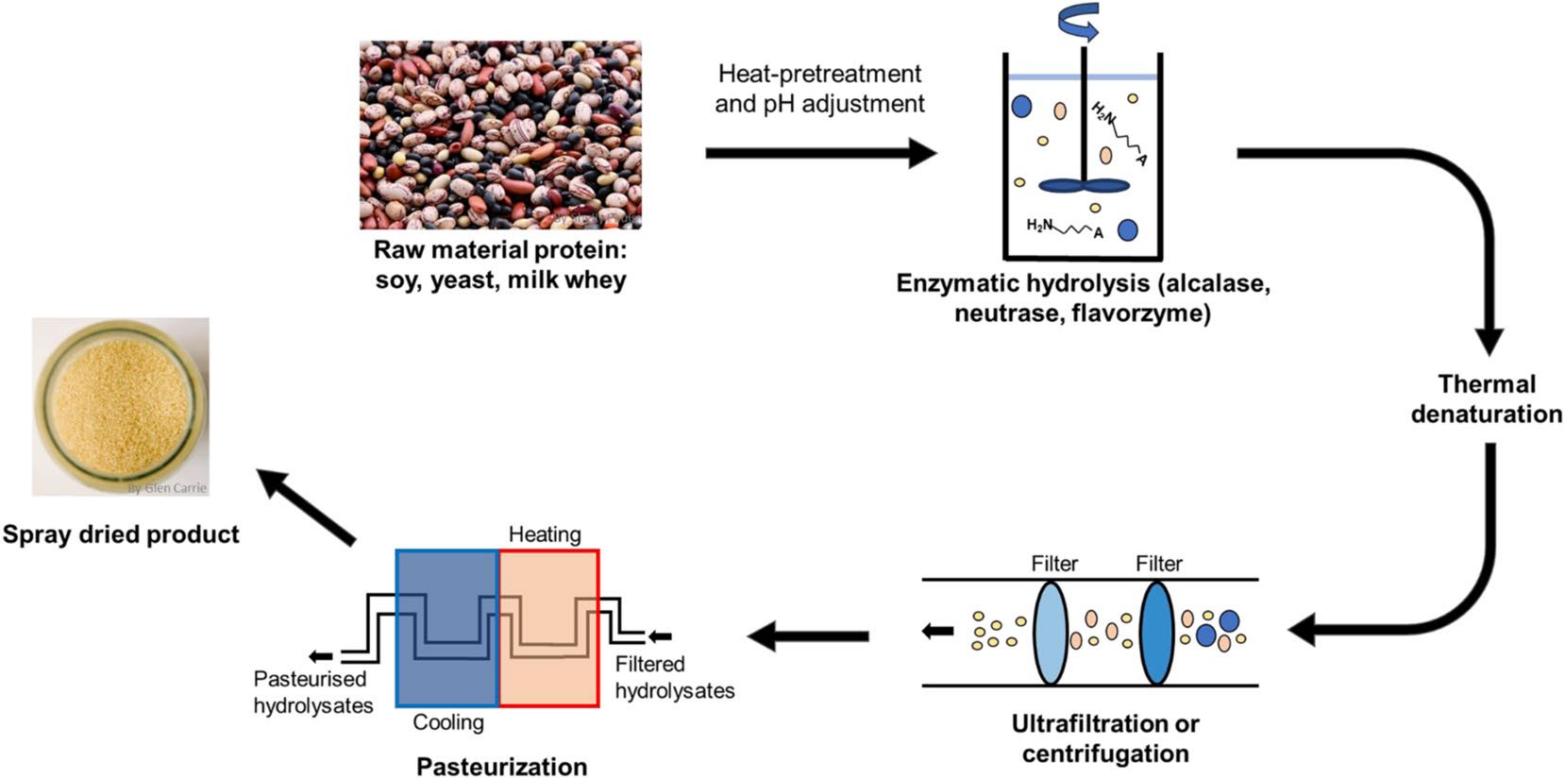
Schematic of hydrolysate production. Raw materials such as soy, yeast, whey or animal proteins were pretreated with heat to break down the protein. After pH adjustment, the mixture is hydrolyzed in a chamber using enzymes, heat or subjected to extreme pH conditions. This process breaks down the protein into smaller components and releases peptides and amino acids into the solution. The degree of hydrolysis is positively correlated with the length of time in this step. Once the desired degree of hydrolysis is achieved, the mixture is again heated to denature the enzymes, followed by centrifugation and ultrafiltration to remove large insoluble particles and endotoxins. The final product is then pasteurized, spray dried and packaged. Photographs are taken from https://Unsplash.com

Hydrolysates are produced from enzymatic digestion and/or acid hydrolysis of micro-organism (e.g., yeast), animal proteins (off-cut, remnants) and plant proteins (e.g., soy, whey, cottonseed), or animal-derived products (e.g., milk) (Fig. 1). These raw ingredients will be pretreated with heat before digestion or hydrolysis by enzymes or chemicals (Petrova et al. 2018). Through the enzymatic/acid hydrolysis processes, a complex mixture of oligopeptides, free amino acids and carbohydrates are released from the raw material (Si and Shang 2020). Therefore, it is necessary to determine which starting materials and the optimal hydrolysis process to obtain the desired proteins and peptides. Upon hydrolysis, the product is filtered by centrifugation and ultrafiltration to remove unwanted materials (Zhang et al. 2019a, b). Finally, the resulting hydrolysate product often undergoes pasteurization to sterilize the product before spray drying and packaging (Fig. 1). Due to lot-to-lot variability in hydrolysate production, quality control (QC) analyses are performed to determine the peptide profile within the hydrolysates. The QC analysis comprises qualitative and quantitative analysis. Under qualitative analysis, monitoring of peptides at different stages of hydrolysis and protease specificity identification is performed by reverse-phase high-performance liquid chromatography (RP-HPLC). On the other hand, under quantitative analysis, cuprimetric assay complemented with total and α-amino nitrogen determination was used to analyze amino acids and peptides since it can predict di- and tri-peptide contents, hence making it more effective in differentiating homogenous hydrolysates and amino acids and poorly hydrolyzed proteins mixture (Silvestre 1997). Additionally, the degree of hydrolysis (DH) is defined as the proportion of the number of peptide bonds being broken in a protein hydrolysate (Rutherfurd 2019). The higher the DH, the greater the number of peptide bonds being cleaved. It can be used as a measure for digestibility, permeability and bioavailability. However, the higher the DH does not equate to higher bioavailability. A study was done on high (48%), medium (27%) and low (23%) DH of whey protein and showed no correlation to the rate of amino acid plasma appearance in humans (Farup et al. 2016).

### Composition of hydrolysates

The compositions of hydrolysates are highly dependent on the starting material used, the bioprocessing methods employed and the degree of hydrolysis (Zhang et al. 2019a ). Hydrolysates comprised a complex mixture of free amino acids, peptides, free acids, carbohydrates that resulted from the partial or complete hydrolysis of proteins (Table 2) (Rungruangsaphakun and Keawsompong 2018). The composition varies depending on the proteases used, hydrolysis conditions such as the duration of hydrolysis, temperature and the source of raw materials (Zhang et al. 2019a, b). During the hydrolysis process, proteins are broken down into peptides by enzymes and/or chemicals by cleaving the peptide bonds between two amino acid residues (Cheong et al. 2018). Peptides are compounds consisting of two to 20 amino acids. They are the major components in hydrolysates and have been widely investigated and reported to possess various biological activities. They are often processed from by-products with low market value and ultimately contribute towards the reduction of waste (Petrova et al. 2018). The molecular size range of the peptides found in hydrolysates is crucial as it dictates their purposes in various applications (Pasupuleti and Braun 2010). For instance, in flaxseed and rice hydrolysates, low molecular weight peptides (1–3 kDa) are generally known to possess better antioxidant effect in comparison to peptides larger than 3 kDa (Zhou et al. 2013; Hwang et al. 2016). The hydrolysate profile should contain mostly peptides with a molecular weight of less than 10,000 Da to be considered a non-protein (protein-free) alternative (Pasupuleti and Braun 2010). In addition to peptides, hydrolysates may contain other molecules such as vitamins, lipids and inorganic acids that could support the growth of animal cells (Table 3).

**Table 2:** Composition of hydrolysates

References for amino acids	Plant-based hydrolysate				Animal-based hydrolysate		Microorganism-based hydrolysate
	Rice bran protein %(*w/w*)	Rapeseed %(*w/w*)	Soy protein %(*w/w*)	Cotton seed %(*w/w*)	Atlantic salmon %(*w/w*)	Chicken hydrolysate %(*w/w*)	Yeast %(*w/w*)
	Amagliani et al. (2017)	He et al. (2013)	Gorissen et al. (2018)	Cheng et al. (2020)	Harnedy et al. (2018), Nesse et al. (2014)	Wu et al. (2020)	Wasserman (1961), Podpora et al. (2016)
Ala	2.25	3.26	2.8	4.1	3.9	5.49	2.67
Arg	2.55	5.23	4.8	13.2	2.85	5.78	1.74
Asn	–	6.4	–	–	4.5	–	–
Asp	2.91	–	–	9.8		8.38	3.69
Cys	0.46	1.13	0.2	–	0.38	0.61	0.47
Gln	–	12.63	–	–	6.83	–	–
Glu	4.39	–	12.4	22.1		13.89	5.77
Gly	1.83	3.82	2.7	4.3	4.2	4.03	1.88
His	0.86	2.58	1.5	3.5	1.2	3.24	0.87
Ile	1.04	2.51	1.9	–	2.33	3.92	1.79
Leu	1.98	5.11	5	3.6	4.13	7.39	2.61
Lys	1.61	4.23	3.4	6.8	3.98	8.12	2.64
Met	0.59	NA	0.3	3.5	1.43	1.87	0.57
Phe	1.25	3.06	3.2	1.2	2.18	3.01	1.75
Pro	1.29	4.34	3.3	7.4	2.55	2.71	2.42
Ser	1.38	3.54	3.4	3.6	2.18	3.35	1.82
Thr	1.31	NA	2.3	5.3	2.33	3.9	1.65
Trp	0.44	0.94	–	–	0.6	0.79	0.49
Tyr	1.06	2.7	2.2	3.2	1.88	2.88	1.38
Val	1.64	3.51	2.2	3	3.08	4.15	2.19
Fat/lipids (reference)	2.73	7	–	17.8 (Bertrand et al. 2005)	0.5	8.38	0.2
References for vitamins	Champagne et al. (2004)		﻿University of Rochester Medical Center (2021)		Nesse et al. (2014)	Pinto e Silva et al. (2008)	Wasserman (1961)
A (retinoid)	0 to 0.00257	–	–	–	–	–	–
B1 (thiamine)	0.00857 to 0.0171	–	4.22 × 10^–8^	–	1.8 × 10^–5^	0.00007	0.0104 to 0.025
B2 (riboflavin)	0.00128 to 0.00307	–	2.53 × 10^–8^	–	1.575 × 10^–5^	–	0.0025 to 0.008
B3 (niacin)	0.191 to 0.356	–	3.46 × 10^–8^	–	3.15 × 10^–4^	–	0.03 to 0.0627
B6 (pyridoxine)	0.00643 to 0.02	–	2.53 × 10^–8^	–	5.025 × 10^–5^	–	0.0023 to 0.004
B9 (folic acid)	0.000286 to 0.001	–	–	–	1.425 × 10^–5^	–	0.0019 to 0.003
B12 (cobalamin)	0 to 0.00257	–	–	–	1.2 × 10^–5^	0.00008	–
Biotin	0.000143 to 0.000357	–	–	–	–	–	0.00011
C (ascorbic acid)	0.0186 to 0.0929	–	–	–	4.275 × 10^–3^	0.0475	–
Choline	0.657 to 1	–	–	–	–	–	–
E (alpha–tocopherol)	0 to 2.86 × 10^–6^	–	–	–	–	–	–
Inositol	2.86 to 5.71	–	–	–	–	–	–
p–Aminobenzoic acid	0.000464	–	–	–	–	–	0.0015 to 0.004
Pantothenic acid	0.0143 to 0.0436	–	1.69 × 10^–8^	–	–	–	0.0072 to 0.0086

“–” indicates no available data

**Table 3:** Hydrolysates improve the growth and productivity of cell lines

Hydrolysate used	Cell type	Purpose	Effects	References
Plant peptones	CHO-320 (a CHO K1 clone)	Human interferon-gamma production cells	Improves cultivation and productivity	Burteau et al. (2003)
Yeast hydrolysate	CHO	Human beta-interferon production	Higher productivity with equivalent glycosylation	Spearman et al. (2014)
Yeast hydrolysate	rCHO (recombinant CHO)	Human thrombopoietin (hTPO) production	Higher cell growth and hTPO production by 11.5 times	Sung et al. (2004), Mosser et al. (2013)
Rice protein hydrolysate	CHO-320	Interferon-gamma production	Protection against oxidation stress from hydrogen peroxide	Mols et al. (2004)
Rice protein hydrolysate	Human HepG cells	Cell-based bioassays for food antioxidant activity analysis	Protection against oxidation stress from hydrogen peroxide	Zhang et al. (2016)
Soy peptones (CoyA2SC, SoyE-110)	CHO DG44	Testing cell model	Improved cell production	Davami et al. (2015)
Yeast, soybean and Ex-Cell CD (chemically defined hydrolysate replacement)	CHO	mAb production	Increased mAb titer and specific productivity	Ho et al. (2016)
Wheat hydrolysate			Improved cell viability but not productivity	
Yeast and soybean hydrolysates			Affected the distribution of galactosylated glycans	
Ex-Cell CD			Maintained glycan profile	
Yeast extract	CHO	Fc-fusion protein production	Improved Fc-fusion protein productivity	Hu et al. (2018)
Yeast extract and peptones	CHO-AMW	Recombinant lgG1 anti-human RhD mAb production	Improved maximal cell density by 70% & IgG production by 180%	Mosser et al. (2013)
Silk sericin hydrolysate (from the waste of silk processing)	CHO and HeLa cells	Testing cell model	Improved cell growth and proliferation	Zhang et al. (2019b)
Chlorella vulgaris extract	CHO-K1 and MSC	Protein expression and steam cell phenotype in MSC	Promoted the growth of CHO and MSC and increased in protein expression in CHO	Ng et al. (2020)
Rapeseed cakes	CHO-C5	Testing model	Improved growth of CHO at hydrolysis degree of 5 to 30%	Chabanon et al. (2007)
Rapeseed	Insect Sf9 cells	expression of recombinant proteins from baculovirus expression system	Promoted the growth of insect Sf9 insect cells in serum-free media	Deparis et al. (2003)
Lactalbumin hydrolysate	Mouse Swiss 3T3 cells	DNA stimulation synthesis	Enhances release of plasminogen activator and stimulates DNA synthesis of mouse Swiss 3T3 cells	Chou et al. (1979)
Kabuli type chickpea	Monocytic THP-1 cells	Tools for investigating monocyte structure and function in both health and disease	Supports the growth of THP-1 cell line in the absence of serum	Girón-Calle et al. (2008)
Wheat gluten protein hydrolysates	Primary human monocytes	Involved in inflammatory and anti-inflammatory processes during an immune response	Leads to potent anti-inflammatory and atheroprotective properties	Montserrat-de la Paz et al. (2020)
Tryptone N1	HEK293 EBNA cell line	Production of Tie2 ectodomain	Leads to a twofold increase in volumetric SEAP (secreted alkaline phosphatase) productivity	Pham et al. (2005)
Bonito hydrolysate	CHO	Anti-human IL-6 receptor antibody production	Leads to a 2.2-fold increase in antibody concentration after 7 days of fed-batch culture	Goto et al. (2008)

### Hydrolysates can enhance biotherapeutic protein quality and yield

The use of hydrolysate in animal cell culture can be dated back to as early as the 1970s. It serves two main purposes in animal cell culture; first being a serum substitute for enhancing cell growth, and second as a stimulant for protein production to improve product titer and quality.

As shown in Table 3, hydrolysates have been used extensively not only to improve cell proliferation or as supplements for cells to transition into a serum-free culture, but also to improve the glycosylation profile of recombinant protein produced by CHO. In CHO cells, Spearman et al*.* (2014) showed that soy hydrolysate promotes cell growth but did not increase protein productivity; while yeast hydrolysate reduces cell growth, but achieved a comparable level of protein productivity and glycosylation profile as an animal-based hydrolysate. This suggests that yeast hydrolysate would be a good alternative to animal-based hydrolysate if the quality of the protein produced is the most important attribute. In a more recent study, cottonseed-derived hydrolysate enhances the galactosylation of CHO-S-RTX and CHO-EG2 cells, which in turn improves the product quality (Obaidi et al. 2021). Furthermore, the glycosylation profile of the protein produced by CHO cells is equivalent between yeast and animal-based hydrolysate (Spearman et al. 2014). This study demonstrated that biopharmaceutical manufacturers should consider the use of yeast hydrolysates to improve protein productivity and glycosylation of the product. Apart from being a serum substitute and stimulant for titer improvement, hydrolysate products such as rice protein hydrolysate have been reported to protect CHO-320 and human HepG2 cells against oxidative stress (Zhang et al. 2016), although the mechanisms through which these bioactive peptides enable this protection remain unknown. Different peptide fractions isolated from rapeseed hydrolysates have been used as a source of short-chain peptides with biological properties, such as immunomodulatory and protease inhibitory effects, to stimulate CHO cell growth and increased protein production (Farges et al. 2006).

Besides CHO cell culture, the use of hydrolysates like chickpea and rapeseed in replacing serum has had some successes in supporting the growth of other human cell lines, although this effect could be cell line dependent (Girón-Calle et al. 2008). For instance, a medium containing pea hydrolysate was a good serum substitute for the growth of the THP-1 cell line, but not the epithelial Caco-2 cell line (Girón-Calle et al. 2008) (Table 3). In HeLa cells, serum-free culture has only been recently achieved in cell media containing silk sericin hydrolysate (Zhang et al. 2019b). Once again, the underlying mechanisms governing these effects remain unknown.

Hydrolysates, as a source of free amino acids, are being used as supplements for basal medium, to sustain cell growth and to allow cell adaptation to serum-free culture media. Hydrolysates can enrich the nutritional profile of the media by increasing the stability of glutamine, and processes other beneficial bioactivities (Table 3), leading to enhanced cell viable density (Lobo-Alfonso et al. 2010; Ng et al. 2020). One critical drawback of using hydrolysates as media supplements is that they are undefined, leading to batch-to-batch quality issues. Indeed, it has been reported that different batches of soy hydrolysates from the same manufacturer had opposite effects on CHO cell-mediated mAb production (Richardson et al. 2015). Batches of soy hydrolysates that contain a high level of adenosine and arginine were negatively correlated with antibody titer in CHO cell culture (Richardson et al. 2015). In contrast, batches that contain a high amount of ornithine and citrulline were positively correlated with antibody titer. Citrulline and ornithine are precursors of polyamines that are very important for cell proliferation (Thomas and Thomas 2001; Richardson et al. 2015). Therefore, the effect of varying levels of pro and anti-growth factors in the different batches of hydrolysates should be thoroughly investigated.

The biopharmaceutical industry aims to formulate chemically defined culture media that contains the least number and amount of components required for cell growth and protein production. Although hydrolysates are undefined, the benefit of hydrolysate could still be realized by pre-determining the effect of hydrolysates on the cell line of interest. Furthermore, with advancements in high-resolution analytical techniques, such as chromatographic and mass spectrometric based technologies, the composition of hydrolysates can be better defined and sources of variability identified.

### Hydrolysates as supplements for cultured meats

Cultured meat (CM) is an alternative meat source that is obtained from animal cells grown in vitro rather than from animal slaughter. The purpose of having CM is mainly driven by animal welfare, environmental and sustainability concerns (Tuomisto and Teixeira de Mattos 2011). It has been discovered that animal farming contributes 18% of greenhouse gases (Steinfeld et al. 2006) and wastewater discharge from slaughterhouses contained a large amount of organic substances like fats, blood and proteins that leads to environmental pollution (Njoya et al. 2019).

Akin to biotherapeutics production, cultured meat production aims to achieve high-yielding culture producing the best quality meat. To achieve such optimal processes, exploration of different types of food-grade ingredients that can be used as growth-promoting agents for a tailor-made serum-free medium is required. O’Neill et al. (2021) have covered in a review on the considerations for the development of culture media that is cost-effective and free of animal ingredients for the production of cultured meat by replacing serum with hydrolysate. Currently, a few studies have described the use of hydrolysates as a media supplement for CM production. Tuomisto and Teixeira de Mattos (2011) reported on the use of *Cyanobacteria* hydrolysate to support muscle cell growth. However, the potential cytotoxicity and safety of microbe-based hydrolysate used has not been thoroughly tested and remains a safety concern. In another study, pork plasma digested with Alcalase was shown to restore bovine skeletal muscle cell function and enhanced cell growth (Andreassen et al. 2020). However, the use of animal-derived hydrolysate for CM production is counterintuitive to the purpose of being slaughter-free meat itself. It is more preferable to use plant- and yeast-based hydrolysates that have been widely used in CHO cell cultivation (Table 3). Hence, the usability of plant- and yeast-based hydrolysates should be thoroughly investigated in cultured meat cultivation as it provides a more economical and potentially safer option.

## Bioactivities of hydrolysates

Hydrolysates consist of several components that aid in cellular survival and growth. Peptides are the major ingredient in hydrolysates mixture that possess beneficial bioactivity properties such as anti-apoptosis, antioxidant, immunomodulatory effect and antibacterial properties. Furthermore, it is valuable and of great interest in understanding the underlying biological functions of the peptides in hydrolysates as it helps to identify specific pathways taken by the cells to regulate these unique bioactivities. In addition, the information could also provide important factors for better media design.

### Anti-apoptotic effects

Apoptosis is an orderly process consisting of several different biochemical reactions that leads to cellular characteristic changes and cell death. This process could be mitigated through the utilization of hydrolysates. It has been discovered that an animal-based protein hydrolysate, Primatone RL, contains factors that have anti-apoptosis properties (Schlaeger 1996). In addition, several other plant-based hydrolysates have the potential to support cellular growth and survival (Franěk et al. 2000). However, the specific peptide sequences that are responsible for these anti-apoptotic properties are still unknown.

### Anti-oxidation properties

Antioxidants are molecules that can prevent or minimize damage to cells caused by free radicals and reactive oxygen species. It has been found that hydrolysates derived from soymilk have a higher level of antioxidants as compared to raw soybean-derived hydrolysates due to the presence of anti-oxidative bioactive amino acids (tyrosine, methionine, histidine, lysine and tryptophan) through food processing (Singh et al. 2014). Other than these amino acids, hydrophobic and cysteine amino acids have antioxidant properties, with the sulfhydryl group of cysteine amino acids acting as a radical scavenger to protect cell tissue from oxidative stress (Kim et al. 2020). However, whether the anti-oxidative properties of hydrolysates can be solely attributed to these amino acids, or are in part due to the presence of even more potent antioxidants remains largely unknown.

### Immunomodulatory effects

Despite the prominent rise of cell therapy, where the engineered immune cell is the product, only a handful of studies researched into the immunomodulatory effects of hydrolysates on the cell. Immunomodulatory peptides in hydrolysates have the potential to bind to the innate and adaptive immune receptors on the cell’s surface, affecting proliferation, cytokines and/or antibody production (Möller et al. 2008; Kiewiet et al. 2018 ). Some hydrolysates and their derivatives were tested for their direct or indirect effects on immune cells activation or proliferation. For instance, in an ex vivo assay using primary mononuclear cells, lactoferrin was shown to reduce cytokine release (Möller et al. 2008), while stimulating the cell proliferation (Möller et al. 2008). In other cases, caseins (and digests), a milk protein, were shown to increase T lymphocytes proliferation (Möller et al. 2008). Despite the limited information available, it has been shown that specific amino acids such as glycine, valine, leucine, proline, glutamine and tyrosine possess immune-modulatory functions (Chalamaiah et al. 2018; Kiewiet et al. 2018 ).

### Antibacterial properties

Several peptides found in hydrolysates possess antibacterial properties and have been reported as potential alternatives to overcome bacterial resistance to conventional antibiotics (Farhana et al. 2019). For instance, peptides containing basic amino acids in cottonseed hydrolysates have been reported to possess such properties (Song et al. 2020). In another study, the antimicrobial properties of chia seed hydrolysate were identified to be derived from peptides containing cationic and hydrophobic amino acids with a consensus sequence of GDVIAIR, or having the amino acid K as either the N- or C-terminal or both (Aguilar-Toalá et al. 2020). Interestingly, it has also been found that the degree of antimicrobial activity from hydrolysates greatly depends on the enzyme used during the hydrolysis process (Tovar-Jimenez et al. 2017). Tovar-Jimenez et al. (2017) showed that hydrolyzing bovine whey protein with the aspartyl protease Eap1 enzyme, as opposed to trypsin and chymotrypsin, resulted in a hydrolysate product with markedly higher antimicrobial activity. Other antimicrobial peptides have been described in egg and camel whey hydrolysates (Al-Mohammadi et al. 2020; Wang et al. 2020). Thus, hydrolysates could be potentially be employed as antimicrobial agents in food and therapeutic applications.

## Contaminants and by-products of hydrolysate

Since hydrolysate is a processed product, contaminants could be present in the raw material used or generated during the hydrolysis process. For example, specific yeast peptides from yeast hydrolysate and beta-glucan particularly (1,3)-*β*-glucan in the cell wall from yeast and plant-based hydrolysates are potentially immunogenic (Jiang et al. 2011). In addition, hydrolysates derived from microbes may contain high levels of lipopolysaccharide (LPS), an endotoxin that is difficult to completely remove from the final product (Lobo-Alfonso et al. 2010). Hydrolysates that are derived from Gram-negative bacteria like *Escherichia* coli would likely contain a high level of LPS contamination, which may require a more stringent post-processing procedure for LPS removal (Xu et al. 2019).

Apart from contaminants, by-products from hydrolysates could also affect cell growth. A recent study has shown significant growth inhibition of the mAb-producing CHO-K1 GS-knockout and CHO-DG44 cells due to the accumulation of tryptophan in the culture (Alden et al. 2020). In the same study, the tryptophan-derived metabolite, 5-HIAAId (5-hydroxyindolacetaldehyde), has been shown to have a detrimental effect on cell growth (Alden et al. 2020). This observation is further supported by a separate study investigating the effects of increased levels of tryptophan by-products and riboflavin degradant (lumichrome), both of which are detrimental to cell growth (Zang et al. 2011). The author explained that high levels of the tryptophan-derived metabolite were a consequence of tryptophan degradation due to light exposure during the culture media preparation process (Zang et al. 2011). The presence of contaminants and by-products in hydrolysates is indeed a substantial drawback for most commercially available low-cost hydrolysates. Understanding how these contaminants are generated and how they can be removed from the final hydrolysate product would be essential to promote their use as animal serum alternatives for biomanufacturing.

## Working towards a chemically defined media with hydrolysates

With the increased interest in using hydrolysates in cell culture as either a feed supplement or a component of a complete culture media formulation, the undefined nature of hydrolysates is a push back to developing a chemically defined substitute. Despite that, the nutritional profiles and bioactivities in hydrolysates are worth pursuing as serum alternatives. Two key challenges need to be addressed for hydrolysates to be more widely used in culture media formulation. Firstly, efficient methods to fractionate hydrolysate into individual components or classes of components must be developed. Liquid chromatography (LC) could be used to separate the complex mixture into multiple fractions. These fractions can then be studied to support cell growth and protein production. At this stage, the identity of the active components and in which LC fractions are unknown, but fractionating these components could eliminate toxins and other unwanted effects from the culture.

Secondly, the identification of active components in those fractions using high-resolution analytical platforms such as mass spectrometry (MS) can be performed. These platforms are specialized in the detection of low molecular weight compounds (< 3 kDa), which potentially include metabolites, oligonucleotides, or larger molecular weight compounds such as protein and peptides. In the past 5 years, there has been significant interest in the use of metabolomics platforms in food sciences to better understand how nutrition contributes to human health. The workflow of identifying the peptides in hydrolysates are: (1) peptides are separated on a column by LC; (2) their masses are analyzed on a high-resolution mass spectrometer (MS); and (3) fragmentation of each peptide is carried out to acquire the amino acid sequence (MSMS). Typically, the acquired mass spectra can then be matched against a database to deduce the identity of the peptide. However, the majority of peptides in hydrolysate comprises less than seven amino acid residues. This presents a major challenge in data analysis as database searches may not provide a unique protein sequence, but numerous matching results (hits). This challenge can be overcome by using high collision energy to dissociate peptides (also known as high collision dissociation, HCD) during fragmentation, and using high-resolution mass spectrometers such as the Orbitrap™ (Hu et al. 2005) and time-of-flight (TOF). More importantly, HCD can generate immonium ions that provide a wealth of information for peptide composition. In addition to immonium ions, the neutral losses from the side chains of peptides can provide a more detailed analysis of peptide composition (Zhang et al. 2019c).

**Fig. 2:**
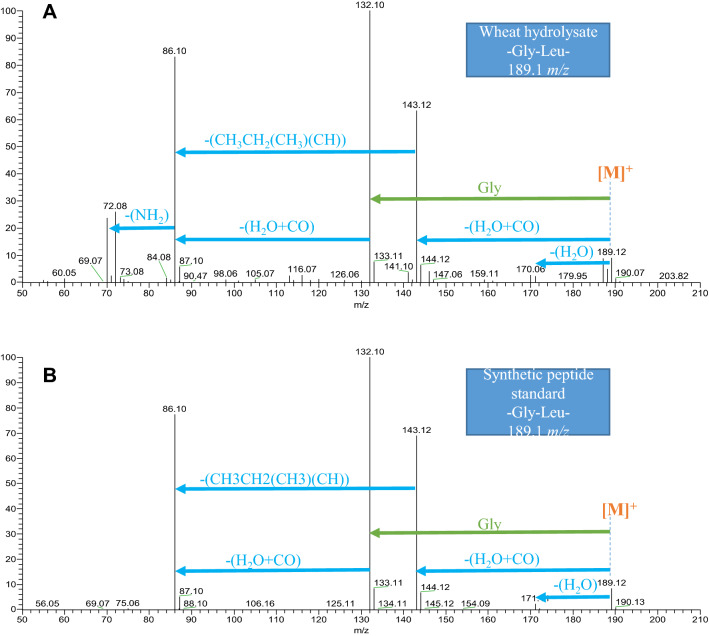
A schematic of MSMS spectra of a protonated short-chain peptide, -Gly-Leu-. These MSMS spectra were acquired on an Orbitrap instrument using collision energy of NCE 10%, showing fragmentation including neutral losses (in blue) and amino acid residue (in green). In this example, as for a dipeptide, only one peptide bond was broken, i.e., the most intense ion in the spectra, 132 m*/z*, loss of 57 from the molecular ion [M]^+^ gives a glycine. This peptide was identified in wheat hydrolysate (**A**) and as compared to a synthetic peptide (**B**), both spectra are identical

Furthermore, positively charged peptides are the most frequently studied peptides in sequencing in comparison to its counterpart, the negatively charged peptides. For many years, researchers have been investigating deprotonated peptides and described the fundamental backbone cleavages and the uniqueness in side-chain fragmentation depending on their specific side-chain structure of amino acid (Bowie et al. 2002). A study performed by Liang and colleagues investigated the side-chain neutral losses of deprotonated di- and longer chain peptides (3–6 residues) and explained how the relative abundance of certain neutral losses can estimate the specific amino acid residues (Liang et al. 2018). This information greatly assists in peptide annotation and confirmation of specific amino acid residues in peptides via either de novo sequencing or matching to a spectral library in the NIST Tandem (MS/MS) library. An example of how a dipeptide is annotated is shown in Fig. 2.

Once the peptide sequence is confirmed, the sequence can then be used to search against a database for its specific functionality, if any. There are approximately 70 peptide databases in the literature where researchers can search a particular peptide with its specific bioactivity (Minkiewicz et al. 2019). The publicly available BIOPEP-UWM database, which is fully curated, is a popular tool for bioactive peptides, especially those derived from foods (Minkiewicz et al. 2019). As of April 2021, the BIOPEP-UWM database contained 740 proteins, 4301 bioactive peptides, 135 allergenic proteins and 492 sensory peptides and amino acids. Another manually curated and publicly available peptide database is called PlantPepDB, specifically focussed on plant proteins, which currently consists of 3848 plant-derived peptides (2821 are experimentally validated) categorized according to their function (Das et al. 2020).

The identification of the active components in hydrolysates could be the first step in the development of such proteins/peptides synthetically or purified from the raw materials. This will be the first of many steps in replacing animal serum with hydrolysates in cell culture. In addition to identifying the active components in hydrolysates, developing processes that can reduce lot variability through better quality control is also important. Efforts to reduce lot-to-lot variation falls between the researcher and the manufacturer; the former could provide novel processes in the detection of components that contribute to product variation, and the latter can help to reduce variation at the point of manufacturing (Thompson and Chesher 2018). In most cases, the lot-to-lot variation is evaluated based on appropriate acceptance criteria such as biological variation outcomes and requirements. Therefore, challenges faced by both researchers and manufacturers are vast not only in defining components in hydrolysates that are bioactive, but also in defining the components that contribute to lot-to-lot variation. A synergetic collaboration between the researcher and manufacturer is valuable and mutually beneficial.

## Conclusion

Although animal serum contains an abundance of essential nutrients and growth factors that support the growth of many cell types, the industry is moving away from the use of animal products. Plant-based hydrolysates could be an alternative to animal serum. Studies have shown that some hydrolysates not only contain amino acids, vitamins and lipids that contribute to the basic ingredients of cell culture media, they also contain beneficial peptides and growth factors that either enhance cell proliferation or improve proteins quality, or both. Furthermore, other properties of hydrolysates such as anti-apoptotic, anti-oxidation, antibacterial, antidiabetic properties as well as immunomodulatory effects cannot be ignored. However, due to the complexity of hydrolysates, it is crucial to understand the advantages as well as challenges of using them as media supplements, such as the risk of contamination or harmful by-products that are present. Full characterization of hydrolysates, together with an in-depth analysis of the spent culture media can be used to identify its critical components. We propose the use of liquid chromatography together with advanced proteomics and metabolomics techniques to identify individual components or groups of components and investigate their functions in a model cell line (e.g., CHO or HEK293). This iterative process would improve our understanding of the relationship between the critical components and specific functionality, an essential step towards the formulation of serum-free chemically defined media. Hydrolysates have the potential to play a much bigger role in the ever-increasing demand for cell culture media for the production of biotherapeutics products, cultured meat, as well as in the production of new modality biotherapeutics such as cell and gene therapy products.

## Data Availability

Data and materials are publicly available from publications as described in references.

## Abbreviations

BHK: Baby hamster kidney
CAGR: Compound annual growth rate
CHO: Chinese hamster ovary
CM: Cultured meat
DH: Degree of hydrolysis
DHFR: Dihydrofolate reductase
FDA: Food and Drug Administration
GS: Glutamine synthetase
HCD: High-energy collision dissociation
HEK293: Human embryonic kidney cell 293
LC: Liquid chromatography
LDL: Low-density lipoproteins
LPS: Lipopolysaccharide
MS: Mass spectrometry
MSMS: Tandem MS
QC: Quality control
RP-HPLC: Reverse-phase high-performance liquid chromatography
TOF: Time-of-flight
